# The Association of Ischemia Type and Duration with Acute Kidney Injury after Robot-Assisted Partial Nephrectomy

**DOI:** 10.3390/curroncol30110698

**Published:** 2023-10-31

**Authors:** Fabian Obrecht, Christian Padevit, Gabriel Froelicher, Simon Rauch, Marco Randazzo, Shahrokh F. Shariat, Hubert John, Beat Foerster

**Affiliations:** 1 Department of Urology, Kantonsspital Winterthur, 8401 Winterthur, Switzerland; 2 Department of Radiology and Nuclear Medicine, Kantonsspital Winterthur, 8401 Winterthur, Switzerland; 3 Department of Urology, Medical University of Vienna, 1090 Vienna, Austria; 4 Departments of Urology, Weill Cornell Medical College, New York, NY 10065, USA; 5 Department of Urology, University of Texas Southwestern Medical Center, Dallas, TX 75390, USA; 6 Karl Landsteiner Institute of Urology and Andrology, 1090 Vienna, Austria; 7 Division of Urology, Department of Special Surgery, Jordan University Hospital, The University of Jordan, Amman 19328, Jordan; 8 Department of Urology, Second Faculty of Medicine, Charles University, 15006 Prague, Czech Republic; 9 Institute for Urology and Reproductive Health, I.M. Sechenov First Moscow State Medical University, 119435 Moscow, Russia

**Keywords:** partial nephrectomy, robotics, renal cancer, acute kidney injury, ischemia time, selective artery clamping

## Abstract

Background: Acute kidney injury (AKI) after robot-assisted partial nephrectomy (RAPN) is a robust surrogate for chronic kidney disease. The objective of this study was to evaluate the association of ischemia type and duration during RAPN with postoperative AKI. Materials and methods: We reviewed all patients who underwent RAPN at our institution since 2011. The ischemia types were warm ischemia (WI), selective artery clamping (SAC), and zero ischemia (ZI). AKI was defined according to the Risk Injury Failure Loss End-Stage (RIFLE) criteria. We calculated ischemia time thresholds for WI and SAC using the Youden and Liu indices. Logistic regression and decision curve analyses were assessed to examine the association with AKI. Results: Overall, 154 patients met the inclusion criteria. Among all RAPNs, 90 (58.4%), 43 (28.0%), and 21 (13.6%) were performed with WI, SAC, and ZI, respectively. Thirty-three (21.4%) patients experienced postoperative AKI. We extrapolated ischemia time thresholds of 17 min for WI and 29 min for SAC associated with the occurrence of postoperative AKI. Multivariable logistic regression analyses revealed that WIT ≤ 17 min (odds ratio [OR] 0.1, *p* < 0.001), SAC ≤ 29 min (OR 0.12, *p* = 0.002), and ZI (OR 0.1, *p* = 0.035) significantly reduced the risk of postoperative AKI. Conclusions: Our results confirm the commonly accepted 20 min threshold for WI time, suggest less than 30 min ischemia time when using SAC, and support a ZI approach if safely performable to reduce the risk of postoperative AKI. Selecting an appropriate ischemia type for patients undergoing RAPN can improve short- and long-term functional kidney outcomes.

## 1. Background

Partial nephrectomy is the recommended treatment for small renal masses and provides safe oncological results in the case of renal cell carcinoma [1]. In comparison to radical nephrectomy, the nephron-sparing approach preserves postoperative kidney function and decreases the risk of chronic kidney failure [2]. It further appears to reduce the rate of cardiovascular events and overall mortality [3,4]. Robot-assisted partial nephrectomy (RAPN) provides satisfactory perioperative and functional outcomes even for renal masses with moderate to high complexity [5,6]. Renal ischemia during resection is used to control the bleeding and improve visualization during resection. Temporarily clamping all afferent renal arteries is considered as warm ischemia (WI). Depending on the duration of arterial clamping, healthy kidney parenchyma may be damaged due to ischemia [7]. Therefore, it is generally recommended to keep the renal ischemia time as short as possible [8]. Alternative ischemia types have been described to reduce ischemic damage. Selective artery clamping (SAC) is a technique where areas of healthy renal parenchyma remain perfused by clamping segmental arteries that supply the tumor area [9]. When using the zero ischemia (ZI) approach, the clamping of afferent renal arteries during resection is completely avoided [10].

Acute kidney injury (AKI)—regardless of etiology—can aggravate preexisting or induce chronic kidney failure [11]. AKI after partial nephrectomy leads to a higher risk of long-term renal function decline and has been suggested as a strong surrogate event [12]. Previous studies identified age, male sex, BMI, diabetes, arterial hypertension, a decreased baseline estimated glomerular filtration rate (eGFR), and a high tumor nephrometry score as given risk factors for postoperative AKI [13,14,15]. Modifiable risk factors for postoperative AKI, on the other hand, have not been sufficiently investigated and deserve more attention. Besides encouraging data showing that WI below 20 min and cold ischemia are associated with decreased rates of postoperative AKI [8,16], evidence on SAC and ZI remains limited [17]. Furthermore, the value of the ischemia time using SAC has not been evaluated.

The objectives of this study were to identify optimal ischemia time thresholds for WI and SAC during RAPN and to investigate the association of WI, SAC, and ZI, within the proposed time thresholds, with postoperative AKI.

## 2. Methods

### 2.1. Study Design

We retrospectively reviewed all patients who underwent RAPN for small renal masses at a tertiary referral center between 2011 and 2019. The Cantonal Review Board of Zurich approved the study protocol (no. 2017-01148). We elaborated this observational study according to the STROBE statement for cohort studies [18]

Data collection was based on the electronic case history and surgical video recordings. Patient characteristics collected to assess for potential confounders included patient age, gender, body mass index (BMI), the presence of arterial hypertension or diabetes mellitus, the presence of accessory arteries, Charlson Comorbidity Index, and baseline estimated glomerular filtration rate (eGFR).

Patients who received marsupialization of benign cysts, bilateral or multiple RAPNs, or intraoperative conversion to open radical nephrectomy or partial nephrectomy were excluded. We further excluded patients with single kidneys or evidence of metastasis (Supplementary Figure S1).

### 2.2. Outcome Measurements

The estimated glomerular filtration rate (eGFR) was calculated using the serum creatinine level according to the Chronic Kidney Disease Epidemiology Collaboration (CKD-EPI) formula [19]. AKI was defined according to the Risk, Injury, Failure, Loss, End-Stage (RIFLE) criteria (reduction of >25% compared to preoperative baseline eGFR or >1.5-fold increase compared to preoperative creatinine, both measured during postoperative hospital stay until discharge) [20]. We determined the presence of AKI using the highest creatinine level measured during the postoperative hospital stay. Serum creatinine levels were measured during oncologic follow-up at 3, 6, and 12 months postoperatively and yearly thereafter. A significant long-term eGFR reduction was defined as a loss of eGFR > 25% in comparison to the preoperative, baseline eGFR. This reduction was defined based on the National Institute for Health and Care Excellence guidelines for chronic kidney disease [21].

### 2.3. Surgical Parameters

All RAPN were performed either by the laparoscopic or retroperitoneoscopic approach using the *da Vinci*^®^ surgical system (Intuitive Surgical, Inc., Sunnyvale, CA, USA). Generally, we used a four-arm robotic system with a maximum of two accessory 12 mm trocars. The trocar number and sites were determined at the surgeon’s discretion. After dissection of the arteries, bulldog clamps were used to interrupt blood flow to selected arteries. Once the tumor was resected, we closed the renal parenchymal defect with two layers of sutures. First, the resection area was closed using an absorbable, monofilament suture with clips on both ends to tighten it for hemostasis. After this step, the early declamping of afferent arteries was pursued. Second, we placed absorbable braided sutures for renorrhaphy. Overall, four senior surgeons performed all operations. To ensure standardized surgical techniques in the department, a consensus was reached among the surgeons at the start of the robotics program and during regular staff meetings.

The operating surgeon chose the ischemia type according to personal preference, based on the tumor characteristics, the vascular situation of the affected kidney, and the patient’s medical history. The used ischemia types were warm ischemia, selective artery clamping, and zero ischemia. Warm ischemia was defined as ischemia of the entire kidney by clamping all afferent renal arteries without remaining perfusion [8]. By clamping segmental arteries or higher-order arterial branches or leaving an accessory artery open, the blood perfusion of the tumor area was cut off while parts of the healthy renal parenchyma remained perfused [9,22]. We administered 5 mg of intravenous indocyanine green to confirm ischemia of the tumor area during fluorescence imaging when using SAC. The degree of remaining perfusion was not measured. Zero ischemia was considered a surgical technique in which all afferent arteries were identified but left open during resection [10].

A senior radiologist reviewed the preoperative CT or MRI imaging for the confirmation of accessory renal arteries and verification of the used ischemia type. We individually reviewed all operations’ video recordings regarding the used ischemia type and time. Tumor complexity was assessed through the preoperative CT or MRI images using the R.E.N.A.L. (radius, exophytic/endophytic properties, nearness of tumor to the collecting system or sinus, anterior/posterior, hilar tumor touching the main renal artery or vein, and location relative to polar lines) nephrometry score [23].

### 2.4. Statistical Analysis

We reported continuous and categorical variables using the median with interquartile range (IQR) and frequencies with proportions, respectively. For the comparison of patients’ baseline characteristics between subgroups, the Mann–Whitney U or χ^2^ test was used for continuous and categorical variables, respectively.

We used STATA’s “cutpt” program to define optimal ischemia time thresholds for SAC and WI to predict the risk of postoperative AKI. This program integrates the Youden index and the Liu method to extrapolate cut-off points with optimal sensitivity and specificity. The Youden method maximizes the sum, whereas the Liu method maximizes the product of sensitivity and specificity. It further bootstraps the standard error to estimate the 95% confidence interval for the calculated threshold value.

Univariable and multivariable binary logistic regression analyses were performed to investigate predictors for postoperative AKI. We created a multivariable model for the risk of postoperative AKI by including the extrapolated ischemia types of interest and all significant parameters from the univariable analysis. We calculated the receiver operating characteristic–area under the curve (ROC-AUC) to evaluate the model’s performance accuracy. The clinical net benefit of the model was assessed by decision curve analysis, which can be interpreted as a comparison between the proposed model and the model without ischemia types, giving the percentage of patients saved from AKI after RAPN within a certain threshold probability. Based on the proposed model, we generated a nomogram ready for clinical use to predict the probability of postoperative AKI.

The association of AKI and its risk factors with long-term kidney function was assessed using Kaplan–Meier estimates and Cox proportional hazard regression models. The predefined ischemia types of interest and all significant parameters from the univariable Cox regression analysis were included in multivariable model 1. In multivariable model 2, all variables with *p*-values ≤ 0.06 from the univariable Cox regression analysis were considered. All statistical analyses were conducted using STATA Version 16.0 (StataCorp LLC. College Station, TX, USA). All tests were two-sided and a *p*-value < 0.05 was considered statistically significant.

## 3. Results

Overall, 154 patients who underwent RAPN were included in the analysis (Supplementary Figure S1). The median age was 65 (interquartile range [IQR] 57–75) years). Postoperative AKI was observed in 33 (21.4%) patients. Patients’ baseline characteristics are listed in Table 1.

For the prevention of postoperative AKI, we computed the optimal time thresholds for WI time and SAC time at 17 min (95% confidence interval (CI): 13.3–20.7 min, *p* < 0.001) (Supplementary Figure S2) and 29 min (95% CI: 20.2–37.8 min, *p* < 0.001) (Supplementary Figure S3), respectively. Accordingly, we built five groups to evaluate ischemia types: zero ischemia, warm ischemia ≤17 min and >17 min, and selective artery clamping ≤29 min and >29 min (Table 1). Twenty-one (13.6%) patients received surgery without ischemia. Among 90 (58.4%) patients who had RAPN with warm ischemia, 45 (29.2%) patients were operated with warm ischemia ≤17 min and >17 min, respectively. Selective artery clamping was used in 43 patients, whereas 32 (20.8%) and 11 (7.1%) patients had ≤29 min and >29 min SAC, respectively.

**Figure 1:**
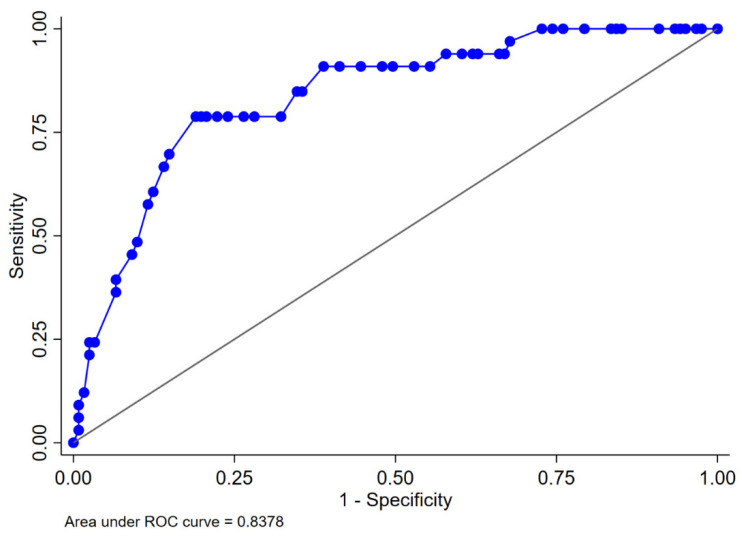
Area under the ROC curve for the proposed multivariable logistic regression model predicting acute kidney injury in 154 patients who underwent robot-assisted partial nephrectomy. ROC: receiver operating characteristic. The multivariable model includes gender, R.E.N.A.L. nephrometry score, and ischemia type.

**Figure 2:**
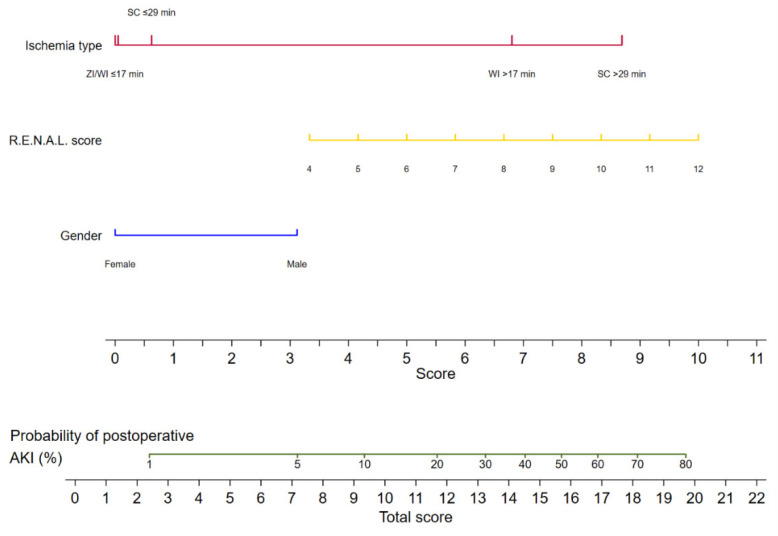
Nomogram predicting the probability of postoperative acute kidney injury for patients undergoing robot-assisted partial nephrectomy. AKI: acute kidney injury; ZI: zero ischemia; WI ≤ 17 min: warm ischemia time ≤ 17 min; SC ≤ 29 min: selective artery clamping ≤ 29 min; WI > 17 min: warm ischemia time > 17 min; SC > 29 min: selective artery clamping > 29 min; R.E.N.A.L. score: radius, exophytic, nearness, anterior, location nephrometry score.

**Figure 3:**
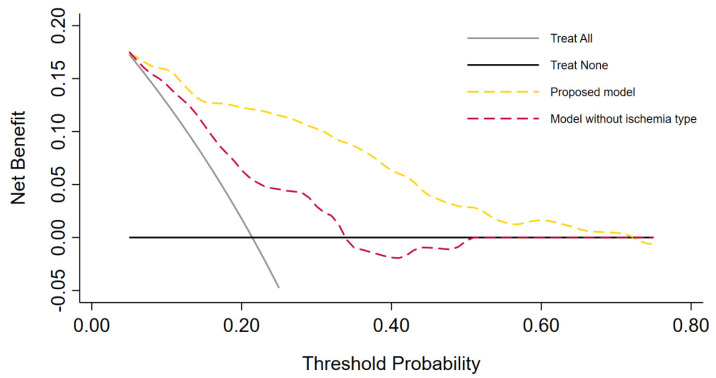
Decision curve analysis demonstrating the clinical net benefit of the proposed multivariable model versus the model without inclusion of ischemia type. Clinical net benefit has an immediate clinical interpretation. The value of 0.12 at a threshold probability of 20% can be interpreted as follows: “Compared to using the model without ischemia type (CNB 0.06) or no auxiliary tool at all, performing RAPN on the basis of the proposed model is the equivalent of a strategy that found 6 or 12 patients per hundred patients saved from postoperative acute kidney injury, respectively”.

The logistic regression analyses for the prediction of postoperative AKI are displayed in Table 2. In the multivariable analysis, female sex (odds ratio [OR] 0.34, 95% confidence interval [CI] 0.13–0.92, *p* = 0.033), zero ischemia (OR 0.10, 95% CI 0.01–0.84, *p* = 0.035), warm ischemia ≤17 min (OR 0.10, 95% CI 0.03–0.37, *p* < 0.001), and SAC time ≤29 min (OR 0.12, 95% CI 0.30–0.47, *p* = 0.002) were independently associated with the occurrence of postoperative AKI. The R.E.N.A.L. nephrometry score could not retain significance (OR 1.33, 95% CI 0.97–1.83, *p* = 0.08). The multivariable model provided high accuracy with an 84% AUC-ROC (Figure 1). Based on this model, we generated a preoperative nomogram to calculate the individual probability of postoperative AKI according to the foreseen ischemia type (Figure 2). Decision curve analysis showed a clinical net benefit of 0.12 at the threshold probability of 20% by applying this model (Figure 3).

During a median follow-up of 19 months, a total of 28 (18.2%) patients experienced a significant eGFR reduction. Cox proportional hazard regression models predicting a significant long-term eGFR reduction are displayed in Table 3. Postoperative AKI was independently associated (HR 7.46, 95% CI 3.42–16.24, *p* < 0.001) with a significant long-term eGFR reduction (Supplementary Figure S4). None of the other included risk factors retained significance.

## 4. Discussion

In this retrospective analysis, we demonstrated that the choice of intraoperative ischemia type during RAPN has profound implications for the occurrence of postoperative AKI, which is one of the strongest prognostic factors for long-term functional outcome. Dependent of the used ischemia type, the ischemia time seems of paramount importance. When RAPNs were performed with ZI, WI below 18 min, or SAC below 30 min, the risk of postoperative AKI decreased by approximately 90%.

When using the WI approach, we found that an ischemia time above 17 min best predicted the incidence of postoperative AKI. The optimal WI cut-off for the purpose of kidney function preservation has been debated for years and was initially suggested to be 30 min [7]. In addition, several studies have recommended that it should remain below 25 min [24,25]. A large observational study identified a WI below 20 min as a strong modifiable, protective factor and showed that each additional minute of WI above 20 min was associated with a significant eGFR decrease [26]. The 20 min threshold is currently considered the common standard, which is in accordance with our proposed cut-off, since the associated 95% CI comprises this mark. However, the studies supporting the 20 min cut-off conducted a categorical, binary analysis by arbitrarily choosing different thresholds [27]. The strength of our analysis is that we extrapolated an ideal cut-off point by analyzing the ischemia time as a continuous variable before implementing it in the multivariable analysis. Our results may explain why prospective trials did not find differences in functional outcomes comparing WI with ZI, while median WI times were between 13 and 19 min among these studies [28,29,30].

We could further point out that SAC below 30 min is equally protective as ZI or WI below 18 min for the occurrence of postoperative AKI in comparison to WI above 17 min. To our knowledge, this is the first ischemia time cut-off proposed for SAC during RAPN. Selective artery clamping allows the partial ischemia of the kidney harboring the tumor and therefore better visualization in bloodless conditions during tumor resection, while other parts of the kidney are still perfused. Under these conditions, the pathophysiological mechanisms for the development of postoperative AKI are not completely clear yet, but it seems that selective ischemia affects the healthy renal parenchyma by inducing an inflammatory response in combination with renal ischemia–reperfusion injury and subsequent cellular damage [31]. Previous studies claimed that the beneficiary effect of SAC or ZI on renal function might only occur during short-term follow-up [17,22,29]. Robust data are lacking since neither individual studies nor meta-analyses comparing different ischemia techniques have taken the ischemia time into account, which is a key factor in assessing different ischemia types [32,33,34]. Under these circumstances, the current literature does not identify a superior ischemia technique [34]. However, a recent study investigating single kidney patients undergoing RAPN revealed no difference in postoperative kidney function between WI and SAC with median ischemia times of 13 and 14 min, respectively [35].

Thirdly, we were able to demonstrate that the ZI approach produced comparable, tending to be slightly better, functional results to WI below 18 min and SAC below 30 min. This tendency can also be observed when inspecting our nomogram. The decisive advantage is that the surgeon is not limited in terms of the ischemia time and can fully avoid damage to healthy renal tissue due to ischemia. Whenever a resection needs more time due to the intermediate or high complexity of the tumor, the ZI approach should be pursued if complete tumor resection is safely feasible. Since its first description, ZI has been shown to be effective even in large and/or endophytic tumors [36,37,38]. However, intraoperative artery clamping provides better visualization of the resection site and the reduction of blood loss. In inexperienced hands, ZI can lead to increased blood loss and higher rates of positive surgical margins. Nevertheless, the long-term results showed that positive surgical margins were well within the warm ischemia series, so the use of ZI should be strongly considered based on the surgeon’s expertise and experience [39].

While most of the supporting evidence for the 20 min WI cut-off relates to its association with mid- to long-term kidney function [26,27], our study focused on postoperative AKI as the primary endpoint, due to the fact that AKI has been suggested as a surrogate endpoint for long-term functional outcomes following RAPN [12,27]. Our results re-affirmed that postoperative AKI increased the risk for a significant long-term eGFR decrease by approximately seven times. Regardless of the etiology, AKI is recognized as a pivotal risk factor for the development of chronic kidney disease [40]. The International Society of Nephrology (ISN) also recognizes its importance as one of the major causes of new-onset chronic kidney disease, end-stage renal disease, and increased healthcare costs in the US [41]. Interestingly, the baseline eGFR in our study had no influence on the occurrence of AKI or on the occurrence of a long-term eGFR reduction, whereas previous large observational studies have shown that patients with a lower baseline eGFR or chronic kidney disease have a higher risk of AKI and a subsequent long-term decline in eGFR [12,15]. This discrepancy and lack of association in our study could be explained by the smaller sample size. However, it should also be taken into account that the association found in these studies with an odds ratio of 1.03 was rather low [15]. Therefore, the overall risk of postoperative AKI is not hugely increased in these patients at 3%. Nevertheless, it is precisely this group that represents the patients most worthy of protection and where surgeons should invest the most effort to avoid AKI.

The natural course of AKI usually shows a return to the baseline eGFR, followed by a deterioration in eGFR influenced by the severity and duration of AKI as well as patient comorbidities [42]. The progression from AKI to chronic kidney disease, which our data affirmed, is thought to arise from a loss in renal reserves and from incomplete or imperfect repair following the acute injury, a so-called maladaptive repair [40]. Through tissue damage induced by AKI, inflammatory cell infiltration, and cytokine release, epigenetic modifications take place that initiate the process of tubulointerstitial fibrosis and therefore reduce the functional renal mass [40]. The long-term eGFR loss is aggravated by the hemodynamic influence through glomerular hypertension on the surviving glomeruli due to the reduced renal mass, leading to glomerulosclerosis [40].

The nomogram that was created could help in assessing the risk of postoperative AKI prior to RAPN. In patients with preexisting chronic kidney disease or a high risk of postoperative AKI, the surgeon can select the ischemia type that corresponds best to the complexity of the tumor. For tumors with low complexity, experienced urologists generally expect ischemia times below 18 min and can safely apply WI. For tumors with higher complexity, however, urologists need to decide whether a time-consuming hilar preparation to dissect the branches of the main renal artery to apply SAC is reasonable to gain more time for a challenging resection. If the surgeon has experience, ZI is another treatment option to prevent AKI in such cases. This nomogram, along with other published predictive tools, could provide assistance for the preoperative decision-making process to reduce the risk of postoperative AKI and thereby long-term kidney function loss according to given risk factors [12,15].

This study has several limitations, such as being single-center with a retrospective design and a small sample size. In addition, the RAPNs were performed by four different surgeons. Despite the institution’s efforts to standardize the surgical techniques, certain differences could have had a confounding influence on the results. However, all surgeons were experienced in partial nephrectomy. The ischemia type was performed at the surgeon’s discretion and therefore selection bias was evident. When looking at the kidney function, there are two main limitations. First, pre- and postoperative kidney function were assessed using serum creatinine levels and the CDK-EPI formula, which is more susceptible to confounders than renal scintigraphy. However, pre- and postoperative scintigraphy were not routinely performed at our department. Secondly, AKI was assessed using serum creatinine levels but not urine output criteria (RIFLE) due to a lack of postoperative data. This might have contributed to missed events of AKI. Furthermore, we were unable to account for the extent of remaining renal perfusion caused by the various selective clamping techniques used, which could have introduced additional bias on its effect. Finally, our nomogram as well as our thresholds for the ischemia time require external validation through large prospective studies before implementation in clinical practice.

## 5. Conclusions

The ischemia type and time during RAPN are of paramount importance for the short- and long-term preservation of renal function. Our analysis confirms the commonly accepted 20 min threshold for WI time, supports the ZI approach if safely performable, and suggests less than 30 min ischemia time when using SAC. We could further re-affirm the detrimental effect of postoperative AKI on long-term renal function. Our nomogram could help surgeons to individually estimate the risk of postoperative AKI and select the optimal ischemia type according to patient and tumor characteristics. External validation and large prospective studies are needed to validate our recommendations and extensively investigate the associations with given preoperative risk factors.

## Figures and Tables

**Table 1:** Patient characteristics.

	Acute Kidney Injury						
	Absent n = 121		Present n = 33		Total n = 154		*p*-Value
	No.	%	No.	%	No	%	
Age [median|IQR]	65	57-74	65	60-75	65	57–75	0.6
Sex							0.041
Male	64	52.9	24	72.7	88	57.1	
Female	57	47.1	9	27.3	66	42.9	
BMI ^1^ [median|IQR]	25	22.3–28.8	27	25–29.9	26	23.3–29.1	0.04
Arterial hypertension							0.3
Absent	79	65.3	18	54.5	97	63.0	
Present	42	34.7	15	45.5	57	37.0	
Diabetes							0.1
Absent	110	90.9	27	81.8	137	89.0	
Present	11	9.1	6	18.2	17	11.0	
Accessory artery							0.7
Absent	85	70.2	22	66.7	107	69.5	
Present	36	29.8	11	33.3	47	39.5	
Charlson Comorbidity Index							0.5
0–2	79	65.3	19	57.6	98	63.6	
3	20	16.5	5	15.2	25	16.2	
>4	22	18.2	9	27.3	31	20.1	
Baseline eGFR ^2^ [median|IQR]	81	68.5–94.1	80	66.3–99.3	81	67.0–94.4	0.8
R.E.N.A.L. score [median|IQR]	7	6–8	8	7–9	7	6–8	0.007
Ischemia type							<0.001
Warm ischemia > 17 min	26	21.5	19	57.6	45	29.2	
Warm ischemia ≤ 17 min	42	34.7	3	9.1	45	29.2	
Selective artery clamping > 29 min	4	3.3	7	21.2	11	7.1	
Selective artery clamping ≤ 29 min	29	24.0	3	9.1	32	20.8	
Zero ischemia	20	16.5	1	3.0	21	13.6	
**Total**	121	78.6	33	21.4	154	100	

^1^ kg/m^2^; ^2^ ml/min/1.7 m^2^; IQR: interquartile range; BMI: body mass index; eGFR: estimated glomerular filtration rate; R.E.N.A.L.: radius, exophytic, nearness, anterior, location nephrometry score.

**Table 2:** Uni- and multivariable logistic regression analyses predicting acute kidney injury after robot-assisted partial nephrectomy.

	Univariable		Multivariable Model	
	OR (95% CI)	*p*-Value	OR (95% CI)	*p*-Value
Age	1.01 (0.98–1.05)	0.6		
Sex				
Male	Ref.		Ref.	
Female	0.42 (0.18–0.98)	0.045	0.34 (0.13–0.92)	0.033
BMI ^1^	1.08 (0.99–1.17)	0.1		
Arterial hypertension	1.57 (0.72–3.42)	0.3		
Diabetes	2.22 (0.75–6.54)	0.1		
Accessory artery	1.18 (0.52–2.69)	0.7		
Charlson Comorbidity Index				
0–2	Ref.			
3	1.04 (0.35–3.12)	0.9		
>4	1.70 (0.68–4.28)	0.3		
Baseline eGFR ^2^	1.00 (0.98–1.02)	0.8		
R.E.N.A.L. score	1.43 (1.09–1.86)	0.009	1.33 (0.97–1.83)	0.08
Ischemia type				
Warm ischemia > 17 min	Ref.		Ref.	
Warm ischemia ≤ 17 min	0.15 (0.03–0.36)	<0.001	0.10 (0.03–0.37)	<0.001
Selective artery clamping > 29 min	2.03 (0.61–9.36)	0.2	1.92 (0.46–8.02)	0.4
Selective artery clamping ≤ 29 min	0.19 (0.38–0.53)	0.004	0.12 (0.30–0.47)	0.002
Zero ischemia	0.09 (0.01–0.56)	0.012	0.10 (0.01–0.84)	0.035
Receiver operating characteristic–area under the curve (ROC-AUC)				0.84

^1^ kg/m^2^; ^2^ mL/min/1.7 m^2^; OR: odds ratio; CI: confidence interval; BMI: body mass index; eGFR: estimated glomerular filtration rate; R.E.N.A.L.: radius, exophytic, nearness, anterior, location.

**Table 3:** Uni- and multivariable Cox regression analyses predicting long-term significant eGFR reduction after robot-assisted partial nephrectomy.

	Univariable		Multivariable Model 1		Multivariable Model 2	
	HR (95% CI)	*p*-Value	HR (95% CI)	*p*-Value	HR (95% CI)	*p*-Value
Age	1.03 (0.99–1.09)	0.1				
Sex						
Male	Ref.					
Female	0.73 (0.34–1.58)	0.4				
BMI ^1^	1.07 (0.99–1.15)	0.1				
Arterial hypertension	1.07 (0.50–2.29)	0.9				
Diabetes	1.50 (0.57–3.97)	0.4				
Accessory arteries	1.07 (0.47–2.43)	0.9				
Charlson Comorbidity Index						
0–2	Ref.					
3	1.85 (0.78–4.39)	0.2				
>4	0.77 (0.28–2.16)	0.6				
Baseline eGFR ^2^	0.99 (0.97–1.00)	0.1				
R.E.N.A.L. score	1.27 (0.99–1.63)	0.06			1.08 (0.83–1.40)	0.6
Ischemia type						
Warm ischemia > 17 min	Ref.		Ref.			
Warm ischemia ≤ 17 min	0.52 (0.19–1.37)	0.2	1.55 (0.45–5.27)	0.5		
Selective artery clamping > 29 min	2.00 (0.74–5.37)	0.2	1.42 (0.52–3.87)	0.5		
Selective artery clamping ≤ 29 min	0.22 (0.05–1.00)	0.05	0.43 (0.09–2.01)	0.3		
Zero ischemia	0.15 (0.02–1.13)	0.065	0.41 (0.05–3.45)	0.4		
Acute kidney injury	7.46 (3.42–16.24)	<0.001	7.05 (2.49–19.98)	<0.001	6.99 (3.10–15.73)	<0.001

^1^ kg/m^2^; ^2^ mL/min/1.7 m^2^; HR: hazard ratio; CI: confidence interval; BMI: body mass index; eGFR: estimated glomerular filtration rate; R.E.N.A.L.: radius, exophytic, nearness, anterior, location nephrometry score.

## Data Availability

The datasets generated and/or analysed during the current study 402 are not publicly available due to restrictions in the ethics approval but are available from the 403 corresponding author on reasonable request.

## Supplementary Materials

The following supporting information can be downloaded at: https://www.mdpi.com/article/10.3390/curroncol30110698/s1, Figure S1: Flow diagram of patients’ selection process. Figure S2. Optimal ischemia time cut-off point for warm ischemia to predict acute kidney injury. Optimal ischemia time cut-off point in 90 patients who underwent warm ischemia robot-assisted partial nephrectomy for prediction of postoperative acute kidney injury. The optimal cutpoint was calculated using STATA’s “cutpt” program, which combines the Youden index and the Liu method to find the point with perfect sensitivity and specificity. The result was bootstrapped to estimate 95% confidence intervals. Figure S3. Optimal ischemia time cut-off point for selective artery clamping to predict acute kidney injury. Optimal clamping duration cut-off point in 43 patients who underwent robot-assisted partial nephrectomy with selective artery clamping for prediction of postoperative acute kidney injury. The optimal cutpoint was calculated using STATA’s “cutpt” program, which combines the Youden index and the Liu method to find the point with perfect sensitivity and specificity. The result was bootstrapped to estimate 95% confidence intervals. Figure S4. Kaplan–Meier estimates for significant eGFR reduction-free survival. Kaplan–Meier estimates for significant eGFR reduction-free survival stratified by postoperative acute kidney injury in 154 patients treated with robot-assisted partial nephrectomy. eGFR: estimated glomerular filtration rate; RAPN: robot-assisted partial nephrectomy; AKI: acute kidney injury.

## References

[1] Ljungberg B., Bensalah K., Canfield S., Dabestani S., Hofmann F., Hora M., Kuczyk M.A., Lam T., Marconi L., Merseburger A.S. (2015). EAU guidelines on renal cell carcinoma: 2014 update. Eur. Urol..

[2] Mir M.C., Derweesh I., Porpiglia F., Zargar H., Mottrie A., Autorino R. (2017). Partial Nephrectomy Versus Radical Nephrectomy for Clinical T1b and T2 Renal Tumors: A Systematic Review and Meta-analysis of Comparative Studies. Eur. Urol..

[3] Thompson R.H., Boorjian S.A., Lohse C.M., Leibovich B.C., Kwon E.D., Cheville J.C., Blute M.L. (2008). Radical nephrectomy for pT1a renal masses may be associated with decreased overall survival compared with partial nephrectomy. J. Urol..

[4] Larcher A., Capitanio U., Terrone C., Volpe A., De Angelis P., Dehó F., Fossati N., Dell’Oglio P., Antonelli A., Furlan M. (2016). Elective Nephron Sparing Surgery Decreases Other Cause Mortality Relative to Radical Nephrectomy Only in Specific Subgroups of Patients with Renal Cell Carcinoma. J. Urol..

[5] Simhan J., Smaldone M.C., Tsai K.J., Li T., Reyes J.M., Canter D., Kutikov A., Chen D.Y., Greenberg R.E., Uzzo R.G. (2012). Perioperative outcomes of robotic and open partial nephrectomy for moderately and highly complex renal lesions. J. Urol..

[6] Hennessey D.B., Wei G., Moon D., Kinnear N., Bolton D.M., Lawrentschuk N., Chan Y.K. (2018). Strategies for success: A multi-institutional study on robot-assisted partial nephrectomy for complex renal lesions. BJU Int..

[7] Shekarriz B., Shah G., Upadhyay J. (2004). Impact of temporary hilar clamping during laparoscopic partial nephrectomy on postoperative renal function: A prospective study. J. Urol..

[8] Lane B.R., Gill I.S., Fergany A.F., Larson B.T., Campbell S.C. (2011). Limited warm ischemia during elective partial nephrectomy has only a marginal impact on renal functional outcomes. J. Urol..

[9] Nohara T., Fujita H., Yamamoto K., Kitagawa Y., Gabata T., Namiki M. (2008). Modified anatrophic partial nephrectomy with selective renal segmental artery clamping to preserve renal function: A preliminary report. Int. J. Urol..

[10] Tanagho Y.S., Bhayani S.B., Kim E.H., Sandhu G.S., Vaughn N.P., Figenshau R.S. (2012). Off-clamp robot-assisted partial nephrectomy: Initial Washington University experience. J. Endourol..

[11] Chawla L.S., Eggers P.W., Star R.A., Kimmel P.L. (2014). Acute kidney injury and chronic kidney disease as interconnected syndromes. N. Engl. J. Med..

[12] Martini A., Cumarasamy S., Beksac A.T., Abaza R., Eun D.D., Bhandari A., Hemal A.K., Porter J.R., Badani K.K. (2018). A Nomogram to Predict Significant Estimated Glomerular Filtration Rate Reduction After Robotic Partial Nephrectomy. Eur. Urol..

[13] Schmid M., Abd-El-Barr A.E., Gandaglia G., Sood A., Olugbade K., Ruhotina N., Sammon J.D., Varda B., Chang S.L., Kibel A.S. (2014). Predictors of 30-day acute kidney injury following radical and partial nephrectomy for renal cell carcinoma. Urol. Oncol..

[14] Kim C.S., Bae E.H., Ma S.K., Kweon S.S., Kim S.W. (2014). Impact of partial nephrectomy on kidney function in patients with renal cell carcinoma. BMC Nephrol..

[15] Martini A., Sfakianos J.P., Paulucci D.J., Abaza R., Eun D.D., Bhandari A., Hemal A.K., Badani K.K. (2019). Predicting acute kidney injury after robot-assisted partial nephrectomy: Implications for patient selection and postoperative management. Urol. Oncol..

[16] Rajan S., Babazade R., Govindarajan S.R., Pal R., You J., Mascha E.J., Khanna A., Yang M., Marcano F.D., Singh A.K. (2016). Perioperative factors associated with acute kidney injury after partial nephrectomy. Br. J. Anaesth..

[17] Paulucci D.J., Rosen D.C., Sfakianos J.P., Whalen M.J., Abaza R., Eun D.D., Krane L.S., Hemal A.K., Badani K.K. (2017). Selective arterial clamping does not improve outcomes in robot-assisted partial nephrectomy: A propensity-score analysis of patients without impaired renal function. BJU Int..

[18] Von Elm E., Altman D.G., Egger M., Pocock S.J., Gøtzsche P.C., Vandenbroucke J.P., Initiative S. (2014). The Strengthening the Reporting of Observational Studies in Epidemiology (STROBE) Statement: Guidelines for reporting observational studies. Int. J. Surg..

[19] Levey A.S., Stevens L.A., Schmid C.H., Zhang Y.L., Castro A.F., Feldman H.I., Kusek J.W., Eggers P., Van Lente F., Greene T. (2009). A new equation to estimate glomerular filtration rate. Ann. Intern. Med..

[20] Bellomo R., Ronco C., Kellum J.A., Mehta R.L., Palevsky P., Acute Dialysis Quality Initiative (ADQI) Workgroup (2004). Acute renal failure—definition, outcome measures, animal models, fluid therapy and information technology needs: The Second International Consensus Conference of the Acute Dialysis Quality Initiative (ADQI) Group. Crit. Care.

[21] National Clinical Guideline Centre (UK) (2014). Chronic Kidney Disease (Partial Update): Early Identification and Management of Chronic Kidney Disease in Adults in Primary and Secondary Care. National Institute for Health and Care Excellence: Clinical Guidelines.

[22] Desai M.M., de Castro Abreu A.L., Leslie S., Cai J., Huang E.Y., Lewandowski P.M., Lee D., Dharmaraja A., Berger A.K., Goh A. (2014). Robotic partial nephrectomy with superselective versus main artery clamping: A retrospective comparison. Eur. Urol..

[23] Kutikov A., Uzzo R.G. (2009). The R.E.N.A.L. nephrometry score: A comprehensive standardized system for quantitating renal tumor size, location and depth. J. Urol..

[24] Rod X., Peyronnet B., Seisen T., Pradere B., Gomez F.D., Verhoest G., Vaessen C., De La Taille A., Bensalah K., Roupret M. (2016). Impact of ischaemia time on renal function after partial nephrectomy: A systematic review. BJU Int..

[25] Funahashi Y., Hattori R., Yamamoto T., Kamihira O., Kato K., Gotoh M. (2009). Ischemic renal damage after nephron-sparing surgery in patients with normal contralateral kidney. Eur. Urol..

[26] Lane B.R., Babineau D.C., Poggio E.D., Weight C.J., Larson B.T., Gill I.S., Novick A.C. (2008). Factors predicting renal functional outcome after partial nephrectomy. J. Urol..

[27] Rosen D.C., Kannappan M., Paulucci D.J., Beksac A.T., Attalla K., Abaza R., Eun D.D., Bhandari A., Hemal A.K., Porter J. (2018). Reevaluating Warm Ischemia Time as a Predictor of Renal Function Outcomes After Robotic Partial Nephrectomy. Urology.

[28] Rosen D.C., Paulucci D.J., Abaza R., Eun D.D., Bhandari A., Hemal A.K., Badani K.K. (2017). Is Off Clamp Always Beneficial During Robotic Partial Nephrectomy? A Propensity Score-Matched Comparison of Clamp Technique in Patients with Two Kidneys. J. Endourol..

[29] Anderson B.G., Potretzke A.M., Du K., Vetter J.M., Bergeron K., Paradis A.G., Figenshau R.S. (2019). Comparing Off-clamp and On-clamp Robot-assisted Partial Nephrectomy: A Prospective Randomized Trial. Urology.

[30] Kaczmarek B.F., Tanagho Y.S., Hillyer S.P., Mullins J.K., Diaz M., Trinh Q.D., Bhayani S.B., Allaf M.E., Stifelman M.D., Kaouk J.H. (2013). Off-clamp robot-assisted partial nephrectomy preserves renal function: A multi-institutional propensity score analysis. Eur. Urol..

[31] Ratliff B.B., Rabadi M.M., Vasko R., Yasuda K., Goligorsky M.S. (2013). Messengers without borders: Mediators of systemic inflammatory response in AKI. J. Am. Soc. Nephrol..

[32] Deng W., Liu X., Hu J., Chen L., Fu B. (2018). Off-clamp partial nephrectomy has a positive impact on short- and long-term renal function: A systematic review and meta-analysis. BMC Nephrol..

[33] Trehan A. (2014). Comparison of off-clamp partial nephrectomy and on-clamp partial nephrectomy: A systematic review and meta-analysis. Urol. Int..

[34] Greco F., Autorino R., Altieri V., Campbell S., Ficarra V., Gill I., Kutikov A., Mottrie A., Mirone V., van Poppel H. (2019). Ischemia Techniques in Nephron-sparing Surgery: A Systematic Review and Meta-Analysis of Surgical, Oncological, and Functional Outcomes. Eur. Urol..

[35] Badani K.K., Kothari P.D., Okhawere K.E., Eun D., Hemal A., Abaza R., Porter J., Lovallo G., Ahmed M., Munver R. (2020). Selective clamping during robot-assisted partial nephrectomy in patients with a solitary kidney: Is it safe and does it help?. BJU Int..

[36] Guillonneau B., Bermúdez H., Gholami S., El Fettouh H., Gupta R., Adorno Rosa J., Baumert H., Cathelineau X., Fromont G., Vallancien G. (2003). Laparoscopic partial nephrectomy for renal tumor: Single center experience comparing clamping and no clamping techniques of the renal vasculature. J. Urol..

[37] Tuderti G., Brassetti A., Mastroianni R., Misuraca L., Bove A., Anceschi U., Ferriero M., Guaglianone S., Gallucci M., Simone G. (2022). Expanding the limits of nephron-sparing surgery: Surgical technique and mid-term outcomes of purely off-clamp robotic partial nephrectomy for totally endophytic renal tumors. Int. J. Urol..

[38] Brassetti A., Cacciamani G.E., Mari A., Garisto J.D., Bertolo R., Sundaram C.P., Derweesh I., Bindayi A., Dasgupta P., Porter J. (2022). On-Clamp vs. Off-Clamp Robot-Assisted Partial Nephrectomy for cT2 Renal Tumors: Retrospective Propensity-Score-Matched Multicenter Outcome Analysis. Cancers.

[39] Brassetti A., Anceschi U., Bove A.M., Prata F., Costantini M., Ferriero M., Mastroianni R., Misuraca L., Tuderti G., Torregiani G. (2023). Purely Off-Clamp Laparoscopic Partial Nephrectomy Stands the Test of Time: 15 Years Functional and Oncologic Outcomes from a Single Center Experience. Curr. Oncol..

[40] Rangaswamy D., Sud K. (2018). Acute kidney injury and disease: Long-term consequences and management. Nephrology.

[41] Waikar S.S., Curhan G.C., Wald R., McCarthy E.P., Chertow G.M. (2006). Declining mortality in patients with acute renal failure, 1988 to 2002. J. Am. Soc. Nephrol..

[42] Goldstein S.L., Jaber B.L., Faubel S., Chawla L.S., Acute Kidney Injury Advisory Group of the American Society of Nephrology (2013). AKI transition of care: A potential opportunity to detect and prevent CKD. Clin. J. Am. Soc. Nephrol..

