# Supplementary figures and images for: The Association of Ischemia Type and Duration with Acute Kidney Injury after Robot-Assisted Partial Nephrectomy
Source: Curr Oncol. 2023 Oct 31;30(11):9634–46. doi: 10.3390/curroncol30110698 (PMC10670720; doi:10.3390/curroncol30110698)

**Supplementary Figure S1:** Flow diagram of patients' selection process.

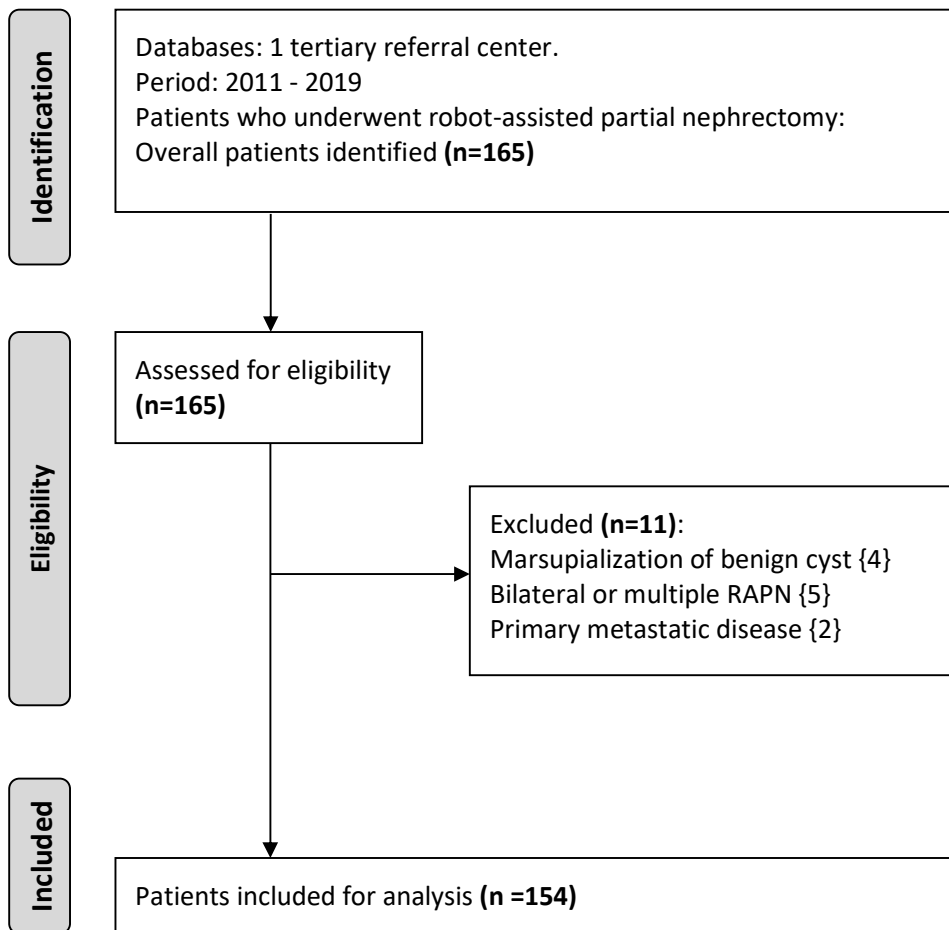

Supplement: Supplementary file 1 [file curroncol-30-00698-s001.zip › Supplementary Figure S1.pdf]

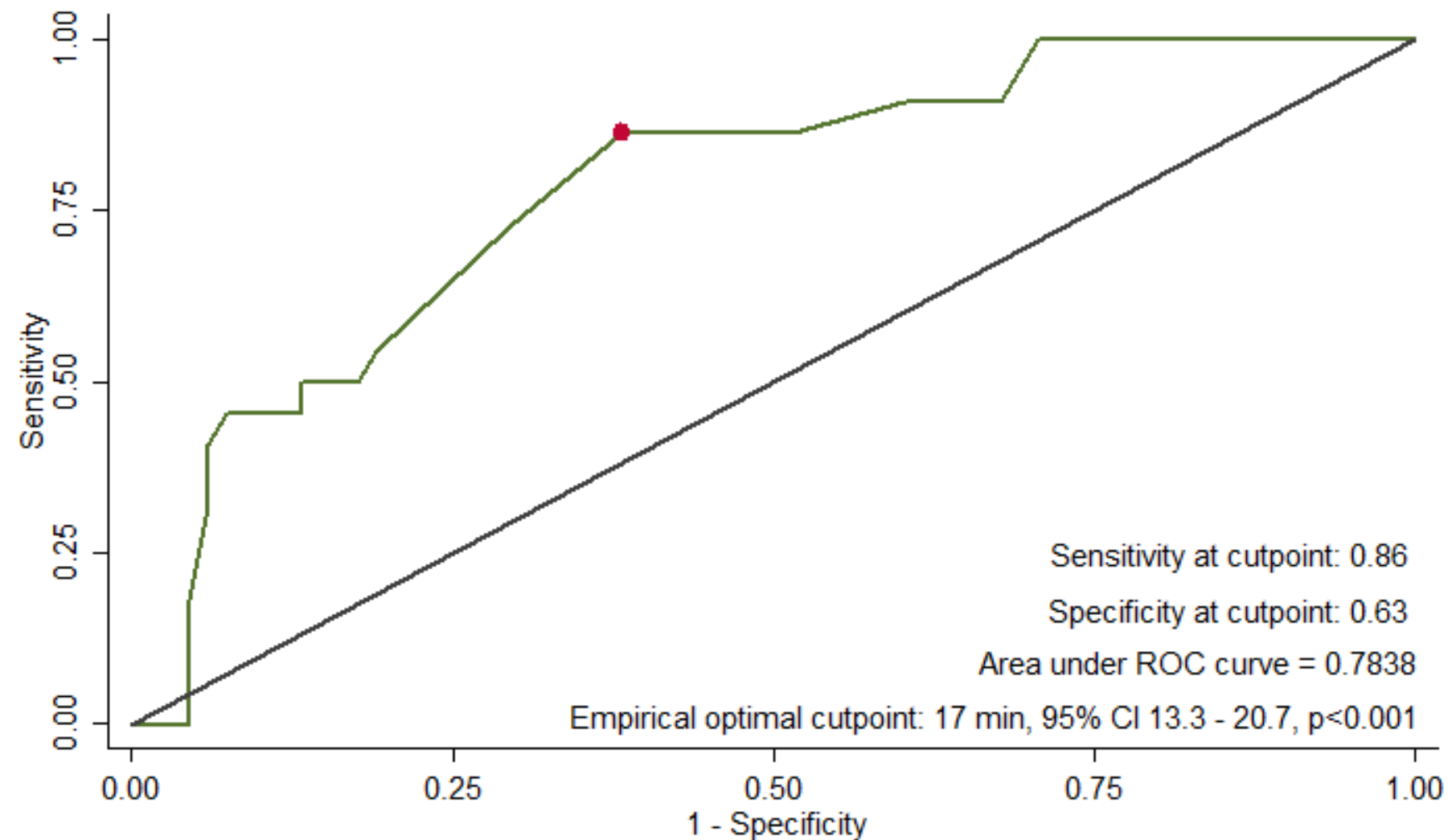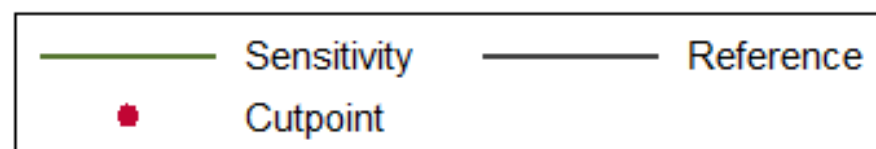

Supplement: Supplementary file 1 [file curroncol-30-00698-s001.zip › Supplementary Figure S2.pdf]

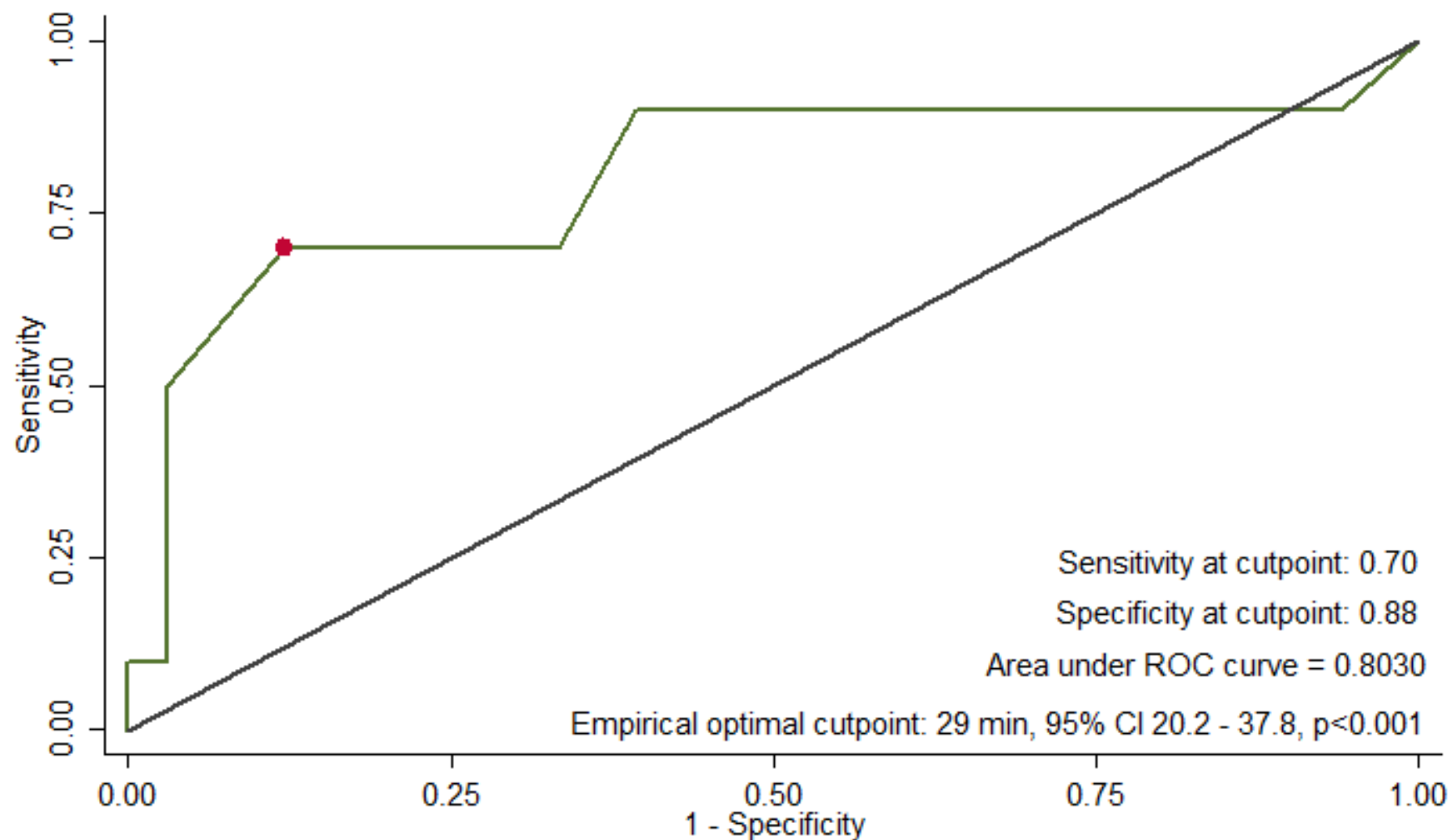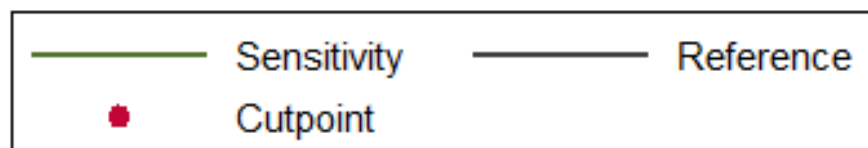

Supplement: Supplementary file 1 [file curroncol-30-00698-s001.zip › Supplementary Figure S3.pdf]

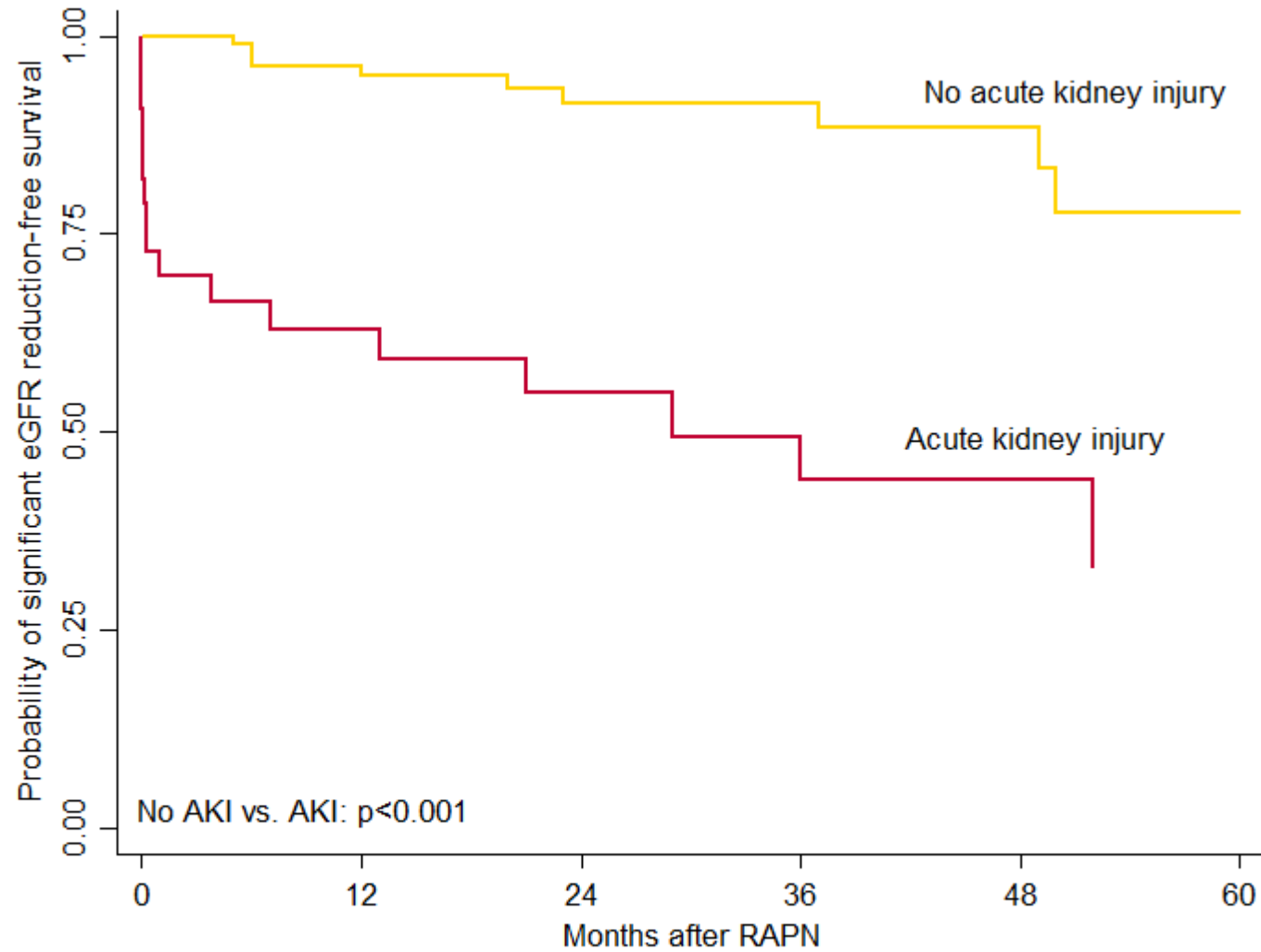

|                        |     |    |    |    |    |   |
|------------------------|-----|----|----|----|----|---|
| Number at risk         |     |    |    |    |    |   |
| No acute kidney injury | 121 | 85 | 47 | 34 | 17 | 9 |
| Acute kidney injury    | 33  | 16 | 12 | 9  | 4  | 3 |

Supplement: Supplementary file 1 [file curroncol-30-00698-s001.zip › Supplementary Figure S4.pdf]
